# Editorial: Transforming food systems: addressing malnutrition and inequality in low- and middle-income countries

**DOI:** 10.3389/fpubh.2025.1747228

**Published:** 2025-12-11

**Authors:** Juliana Souza Oliveira, Margarida Liz Martins, Catarine Santos da Silva

**Affiliations:** 1 Nutrition Unit, Vitória Academic Center, Federal University of Pernambuco, Vitória de Santo Antão, Brazil; 2 Polytechnic University of Coimbra, Coimbra, Portugal; 3 H&TRC—Health & Technology Research Center, Coimbra Health School, Polytechnic University of Coimbra, Coimbra, Portugal; 4 Sports and Physical Activity Research Center, University of Coimbra, Coimbra, Portugal; 5 Research Centre for Anthropology and Health, University of Coimbra, Coimbra, Portugal; 6 Nutrition Department, Federal University of Pernambuco, Recife, Brazil

**Keywords:** food system, food environment, food insecurity, malnutrition, healthy eating, social inequity

Global food systems have undergone profound changes driven by economic, technological, environmental, and cultural transformations that have redefined the way food is produced, distributed, and consumed (1). Increasing industrialization of the food system, coupled with rapid urbanization, and the financialization of global agriculture have turned food into a central commodity within contemporary economic and political power dynamics. This reconfiguration affects not only what people eat but also who has the effective realization of the right to eat well (2). Today, the food system simultaneously produces wealth and inequality, innovation and disease, abundance and hunger. It is, in essence, a mirror of the inequities of the modern world (3, 4). Transforming food systems represents one of the most urgent global priorities to address the intertwined challenges of malnutrition, health inequities, and sustainability in low- and middle-income countries. It has become an urgent global priority to achieve the Sustainable Development Goals, particularly those related to ending hunger (SDG 2), ensuring good health and wellbeing (SDG 3), reducing inequalities (SDG 10), promoting sustainable consumption and production (SDG 12), and combating climate change (SDG 13) (5).

The Research Topic *Transforming food systems: addressing malnutrition and inequality in low- and middle-income countries* brings together evidence, reflections, and experiences that reveal the complexity of this nutritional transition. The selected articles converge on structural challenges faced by low- and middle-income countries: the persistence of hunger and malnutrition, the rise of obesity and non-communicable diseases, and the urgent need to restructure food policies and practices through an equity-oriented lens. In addition to portraying diverse contexts, from Africa to Latin America, from Asia to the Middle East, these studies propose transformative pathways based on the integration of health, sustainability, governance, and social justice.

When revisiting these contributions, it becomes evident that malnutrition in all its forms continues to express the structural inequalities that cross the Global South. Studies conducted in vulnerable contexts, such as among Indigenous populations in Northeastern Brazil (Almeida et al.), pregnant women in Ethiopia (Admass et al.), or rural communities in the Amazon (Orellana et al.), reaffirm that the social, economic, and environmental determinants of health shape nutritional status and food practices. These analyses demonstrate that eating is not merely an individual behavior but the result of complex systems intersecting income, gender, ethnicity, territory, and access to public policies (6, 7). In this sense, transforming food systems requires understanding food as a right rather than an individual choice conditioned by the market (8, 9).

Transformation also requires a critical review of production and supply models. Agricultural and health policies, historically fragmented, have not been able to respond to contemporary challenges (10, 11). The reflections presented in this Research Topic highlight the need for greater integration among sustainable agriculture, food and nutrition security, and social protection policies. Bridging these fields is fundamental to reducing dependency on commodities, strengthening local food systems, and expanding food sovereignty (12, 13). Innovative initiatives such as community food networks and agroecological programs, illustrated by experiences like a new approach to a wicked problem (Schuh et al.) and Randomized community trial to assess nutritional, socioeconomic, and health outcomes of a food forest initiative (Faytong-Haro et al.), demonstrate that transformation is possible when territories become protagonists of their own solutions.

Another central axis of discussion concerns the regulation of food environments. Urban and school contexts, workplaces, and food markets reflect the asymmetry between the marketing of ultra-processed products and policies to promote adequate and healthy eating. Recent studies have shown that the presence of ultra-processed products in daily environments increases the risk of chronic diseases and exacerbates the obesity burden (14, 15). For this reason, a set of regulatory actions, such as taxation of sugary drinks, front-of-pack nutritional labeling, restrictions on child-directed advertising, and strengthening of school feeding programs, is recommended as the foundation of a systemic response (16). These measures protect consumers and reorient production systems, reducing food vulnerability and promoting more conscious and informed choices.

The global syndemic of undernutrition, obesity, and climate change (17) demonstrates that human and planetary health are inseparable. Transforming food systems also means confronting the environmental collapse that sustains them: soil degradation, biodiversity loss, water pollution, and greenhouse gas emissions associated with intensive livestock production and food waste. This convergence of crises calls for state policies grounded in science and in ecological justice. The international agenda from One *Health* to agroecological transition, reinforces that the future of nutrition depends on aligning food, health, and sustainability around a shared collective purpose (18, 19).

The intersection between inequality and nutritional health emerge strongly across the studies in this Research Topic. They reveal that dietary transformations are not homogeneously: marginalized population groups face additional barriers to food access and are more exposed to health risks. Poverty, racism, and gender inequality are central determinants of who eats, what, how, and when (20, 21). Moreover, growing urbanization and labor precarity exacerbate food insecurity among peripheral populations and informal workers (22). These findings reinforce the need for food and nutrition policies to be integrated with broader agendas on poverty reduction, income redistribution, and the promotion of solidarity-based economies (20–22).

The contributions gathered in this Research Topic also highlight the importance of spaces of care and learning, such as schools, health centers, and communities—in promoting healthy eating. These spaces function as privileged *locus* of public policies implementation and innovative pedagogical practices. The literature emphasizes that schools are strategic territories for shaping eating habits, not only for their role in providing meals but also for integrating food education, sustainability, and citizenship (23). Strengthening school nutrition programs, expanding school gardens, and engaging families and educators are concrete pathways to fostering food autonomy from childhood.

Another cross-cutting challenge is the double burden of malnutrition, the coexistence of nutritional deficiencies and excesses within the same territories, families, or even individuals. This paradoxical condition, especially prevalent in middle-income countries, demonstrates that the problem lies not only in what is lacking but also in what is in excess: empty calories, ultra-processed foods, and deep-rooted inequality. Double-duty actions, such as those discussed in Double-duty actions addressing the double burden of malnutrition among adolescents (Zhao et al.), represent a promising approach to integrating prevention and care, overcoming the fragmentation between hunger and obesity agendas (24). When embedded in intersectoral policies and life-course approaches, these actions can produce lasting impacts on population health.

In summary, the articles gathered in this Research Topic demonstrate that transforming food systems is, above all, an agenda of social and environmental justice. It requires rethinking the roles of governments, companies, and civil society in food production and consumption; it demands participatory governance, transparency, and shared responsibility. Science plays a central role in this transition, not only by providing evidence but also by shaping narratives capable of inspiring transformative policies and practices. Food, understood as a cultural, political, and ecological expression, can serve as the axis through which we rebuild bonds among people, territories, and nature.

Transforming food systems, therefore, is a political and ethical act (6, 12, 14). It is about recognizing that access to healthy and sustainable food should not be a privilege but a universal right. It is about restoring to food its sense of belonging and shared meaning. The studies presented here reaffirm that it is not enough to produce more; it is essential to produce with equity, distribute with justice, and consume with awareness. This is the direction pointed out by scientific evidence and by the voices emerging from the Global South: the creation of food systems that are healthy, just, and resilient designed not only to nourish bodies but to sustain lives and future generations.
